# Sensor location affects skeletal muscle contractility parameters measured by tensiomyography

**DOI:** 10.1371/journal.pone.0281651

**Published:** 2023-02-09

**Authors:** Carsten Schwiete, Christian Roth, Christoph Braun, Lukas Rettenmaier, Kevin Happ, Georg Langen, Michael Behringer

**Affiliations:** 1 Department of Sports Sciences, Goethe University Frankfurt, Frankfurt, Germany; 2 Department of Strength Power and Technical Sports, Institute for Applied Training Science, Leipzig, Germany; The Education University of Hong Kong, HONG KONG

## Abstract

Tensiomyography (TMG) is a non-invasive method for measuring contractile properties of skeletal muscle that is increasingly being used in research and practice. However, the lack of standardization in measurement protocols mitigates the systematic use in sports medical settings. Therefore, this study aimed to investigate the effects of lower leg fixation and sensor location on TMG-derived parameters. Twenty-two male participants underwent TMG measurements on the m. biceps femoris (BF) in randomized order with and without lower leg fixation (fixed vs. non-fixed). Measurements were conducted at 50% of the muscle’s length (BF-mid) and 10 cm distal to this (BF-distal). The sensor location affected the contractile properties significantly, both with and without fixation. Delay time (T_d_) was greater at BF-mid compared to BF-distal (fixed: 23.2 ± 3.2 ms vs. 21.2 ± 2.7 ms, *p* = 0.002; non-fixed: 24.03 ± 4.2 ms vs. 21.8 ± 2.7 ms, *p* = 0.008), as were maximum displacement (D_m_) (fixed: 5.3 ± 2.7 mm vs. 3.5 ± 1.7 mm, *p* = 0.005; non-fixed: 5.4 ± 2.5 mm vs. 4.0 ± 2.0 mm, *p* = 0.03), and contraction velocity (V_c_) (fixed: 76.7 ± 25.1 mm/s vs. 57.2 ± 24.3 mm/s, *p* = 0.02). No significant differences were revealed for lower leg fixation (all *p* > 0.05). In summary, sensor location affects the TMG-derived parameters on the BF. Our findings help researchers to create tailored measurement procedures in compliance with the individual goals of the TMG measurements and allow adequate interpretation of TMG parameters.

## Introduction

Skeletal muscle contractility is an important factor influencing athletic performance since efficient cross-bridge cycling is required to generate optimal force [1]. In recent years, tensiomyography (TMG) has emerged as a useful, non-invasive method to assess muscle contractility by measuring the radial displacement of the muscle in response to a standardized, 1 millisecond electrical stimulus. The bipolar electrical stimulus is evoked by two self-adhesive electrodes and is measured by a high-precision displacement sensor placed perpendicularly on the muscle belly [2, 3]. The stimulation intensity is then progressively increased until either maximal radial displacement or the maximal stimulation amplitude is reached. Overall, five primary parameters are derived from TMG: maximum muscle displacement (D_m_) is given in millimeters (mm) and reflects the first peak of the displacement curve; delay time (T_d_) represents the time between the electrical pulse and 10% of the maximum displacement; contraction time (T_c_) reflects the time period from 10% up to 90% of the measurement curve; half-relaxation time (T_r_) is the time between 90% and 50% of D_m_ on the descending curve, and sustain time (T_s_) is the duration of sustained twitch, which is measured between 50% of D_m_ on each side of the twitch curve. As T_c_ represents the time of 10% to 90% of D_m_, it is not possible to interpret T_c_ independently of D_m_, which can hamper the proper interpretation of the actual contraction velocity [4]. Therefore, contraction velocity (V_c_) is calculated as an indirect alternative to T_c_, as V_c_ combines the muscular response to the twitch in temporal and spatial dimensions.

Examining muscle contractility through TMG provides researchers with insights regarding the athlete’s muscle capacities. The measurements are highly reproducible (ICC = 0.92 to 0.97; CV = 2.7% to 4.7%) and correlate with physiological and performance characteristics [2, 5, 6]. Exemplary, TMG parameters correlate with biomarkers of muscle damage in the blood serum following a strength training protocol [7, 8], with maximal oxygen consumption, and maximal cardiac output [9]. In addition, TMG has been applied in the field of body composition [10, 11].

Regarding the individual parameters, there appears to be a relationship between D_m_ and muscle stiffness [12, 13], thus, allowing researchers and practitioners to monitor stiffness through TMG. Moreover, T_c_ is considered to correlate with muscle fiber composition and may be able to provide inferences about the distribution of slow-twitch or fast-twitch fibers [14]. T_d_ can be used to derive implications concerning the reaction speed of the nervous system. García-Manso et al. [15] documented a significant decrease in the T_d_ of the m. rectus femoris following an ultra-triathlon. In addition, T_d_ is associated with the power velocity of muscle fibers and is, thus, related to muscle strength [16]. V_c_ is suitable for detecting functional changes in the mechanical properties of the muscles. Chronic decreases in sprint velocity and change of direction are potentially associated with decreased V_c_, which is, therefore, suggested as a monitoring tool to assess potential impairments when sprinting at top speed [16].

Collectively, TMG offers a non-invasive assessment of an athlete’s muscle characteristics, as well as a straightforward presentation of the measurement results. Nevertheless, the interpretation of the data and its validity are constrained due to a lack in proficient standardization of the measurement protocols [3, 9]. A recent systematic review with meta-analysis by Lohr et al. [4] emphasized a lack of solid evidence for diagnostic accuracy when investigating muscular performance via TMG. For example, parameter variability may be affected by slight changes in joint angle, sensor location or inter-electrode distance [3]. Previous studies have shown that sensor placement on the muscle belly affects D_m_ and T_c_ of different muscles of the trunk, and the upper and lower extremities [17]. In addition, changes in joint angle and, therefore, muscle length affected D_m_, T_d_, and T_c_ in the studies of Ditroilo et al. [18] and Latella et al. [19]. As lower leg movements during the measurement affect the joint angle and muscle length, a fixation of the lower leg might influence the measurement results. While previous research exposed a high variability of TMG measurements due to external factors, the exact impact of sensor position and joint motion during TMG measurements, especially in the lower extremity, remains unclear.

Accordingly, the aim of the present study was to examine the effects of sensor location and lower leg fixation during TMG measurements on the contractile properties of the m. biceps femoris (BF). The BF was chosen as there is already a large body of work regarding the anterior leg muscles, e.g., m. rectus femoris and m. vastus lateralis. The posterior muscle group of the leg, however, is still underrepresented in this research area.

Our hypothesis was that (1) sensor location and (2) lower leg fixation both affect the TMG-derived muscle contractility of the BF. To the best knowledge of the authors, this was the first study to implement TMG measurements at two different measurement sites on the BF. Furthermore, no study to date has investigated the effects of lower leg fixation on the muscle contractility parameters of the BF.

## Methods

### Study design

A single-group randomized-crossover design was used for this study, which was approved by the local ethics committee (ethics committee department 05, Goethe University, Frankfurt am Main, Germany, no.: 2021–17) and conducted in accordance with the ethical standards set by the declaration of Helsinki. Skeletal muscle contractility of the BF of the dominant leg was assessed using tensiomyography (TMG; TMG-BMC Ltd., Ljubljana, Slovenia). The participants lay in a prone position with their ankles placed on the TMG cushion for lower leg measurements, so that the measurement was performed with a knee flexion of about 5° [20, 21]. Two electrodes (self-adhesive, Dura-Stick, 50 ✕ 50 mm) were attached to the disinfected skin with an inter-electrode distance of 5 cm between the facing edges of the electrodes [21, 22]. The TMG sensor was then placed perpendicularly to the skin, with a retraction of approximately 2 cm into its housing. The TMG measurements were performed at two measurement sites and conditions on the BF. The first measurement site was at 50% of the length between the origin (ischial tuberosity) and the insertion (head of the fibula) (BF-mid); the site was marked with a water-resistant pen. To ensure that the mark was correctly placed on the BF, each participant performed an isometric contraction with the hamstrings against the hand of the investigator. Therefore, the participants were instructed, while lying in a prone position, to lift their lower leg until a 90° flexion was reached in the knee joint. Afterwards, the investigator placed his hand against the heel of the participant, who was told to press his heel into the investigator’s hand as strong as possible for 5 seconds. During the isometric contraction, the investigator palpated the BF with his free hand to ensure a correct placement on the muscle belly.

**Fig 1:**
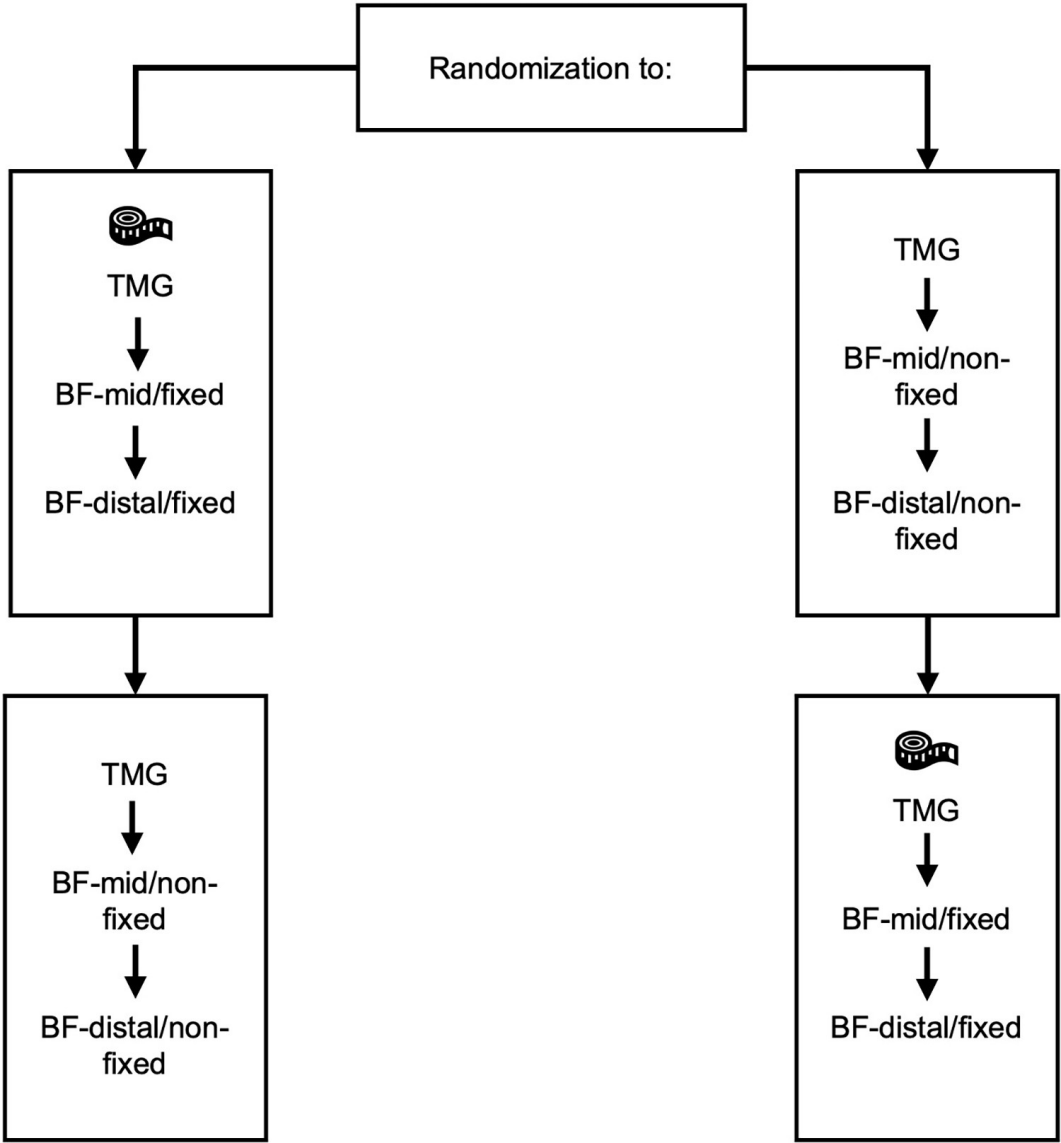
Schematic illustration of the measurement protocol. Participants were randomly allocated to the fixed or the non-fixed measurement condition. After completion of the protocol at both measurement sites on the m. biceps femoris (BF-mid = 50% of BF length (ischial tuberosity to fibula head); BF-distal: 5 cm distal of BF-mid), the subjects received the remaining measurement condition.

The second measurement site was 10 cm distal of BF-mid and was also marked (BF-distal). For BF-distal, the proximal electrode of BF-mid was placed 5 cm below the distal electrode from BF-mid. Hence, the distal BF-mid electrode turned into the proximal electrode for BF-distal, again allowing for an inter-electrode distance of 5 cm. The TMG measurements at both measurement sites (BF-mid and BF-distal) were performed under two conditions: in one condition, measurements were performed without fixation of the lower leg (non-fixed), while the second condition was performed with the participant’s lower leg strapped to the TMG pad using a 25 mm ✕ 2 m non-elastic adjustable strap, which was attached at the Achilles tendon (fixed). Each participant underwent each of the two conditions in a randomized order, as determined by randomizer.org. To mitigate overstimulation of the muscle, a resting interval of approximately 2 minutes was aimed for between the conditions. Ultimately, our study included four measurements in a randomized order per participant, e.g., 1. BF-mid/fixed, 2. BF-mid/non-fixed, 3. BF-distal/fixed, 4. BF-distal/non-fixed (Fig 1).

The TMG measurement itself was performed as follows: starting at 50 mA, the intensity was progressively increased by 10 mA every 30 seconds until maximal displacement or maximal output was reached [10]. Thus, the participants had a maximum of seven stimuli per measurement site. The electrical stimulation consisted of single, monophasic, square wave stimuli, with a duration of 1 ms each. Between each measurement site, a resting interval of 2–3 minutes was included to provide muscle relaxation. For statistical analysis, the measurement curve with the highest D_m_ was used. All other parameters were derived from the same measurement curve. V_c_ was calculated as the mean velocity until 90% D_m_ (V_c_ = 0.9 D_m_/ [T_d_ + T_c_]*1000) was reached [11].

### Participants

Twenty-two male subjects (age: 26.2 ± 3.4 years; height: 182.2 ± 6.7 cm; weight: 80.9 ± 7.7 kg) were recruited to participate in this study. Additional information regarding the body composition and activity profile of the study population is presented in Table 1, as assessed by a 3D Scanner (Scaneca GmbH, Berlin, Germany). Only male subjects were included in this study as sex had been previously reported to influence muscle contractility measured by TMG [4, 23, 24]. The inclusion criteria also required the participants to be between 18 and 35 years of age and to have an average training volume of 2 training sessions per week (strength or endurance based) [25]. Subjects, who were not between the ages of 18 and 35 years, completed less than 2 exercise sessions per week, regularly took medications or suffered from acute lower extremity injuries, were excluded from study participation. Subsequently, the subjects were instructed to refrain from strength or endurance training 48 h before the measurement to avoid measurement distortions. Furthermore, they were informed about the goal of the study, as well as its conduction and every subject voluntarily agreed and provided written and informed consent of participation.

**Table 1:** Baseline characteristics of the study population.

Baseline characteristics	
**Body fat percentage (%)**	14.5 ± 4.2
**Body mass index (kg/m** ^**2**^ **)**	24.6 ± 2.1
**Fat-free mass index (kg)**	20.7 ± 1.5
**Sports per week (h)**	8.0 ± 3.6
**Resistance training per week (h)**	3.3 ± 3.0
**Endurance training per week (h)**	1.6 ± 1.5
**Team sports per week (h)**	3.2 ± 4.2

Data presented as means ± standard deviations.

### Statistical analysis

Prior to statistical analyses, all data were checked for outliers and normal distribution using Boxplots and Shapiro-Wilk’s test of normality, respectively. A two-way repeated measures ANOVA using planned simple contrasts was performed to check for differences between the measurement sites (BF-mid vs. BF-distal) and conditions (fixed vs. non-fixed). Bonferroni correction was applied to impede alpha error accumulation. Given their poor within-subject reliability, T_s_ and T_r_ were only presented via descriptive statistics. Pearson’s correlation was carried out to investigate the strength of the relationship between the variables, while Bland and Altman’s limits of agreement (LOA) were calculated to determine the degree of agreement (bias and LOA) between conditions [26]. Linear regression was employed to detect proportional bias. All tests were based on a 5% level of significance. Data are presented as means ± standard deviations, including Cohen’s d_z_ if adequate.

## Results

Table 2 shows the descriptive values of all TMG-derived parameters across the measurement conditions (fixed vs. non-fixed) and sites on the BF (BF-mid vs. BF-distal).

**Table 2:** Descriptive overview of all TMG-derived parameters.

Measurement condition	Fixed		Non-fixed	
**Measurement site**	**BF-mid**	**BF-distal**	**BF-mid**	**BF-distal**
D_m_ (mm)	5.3 ± 2.7*	3.5 ± 1.7	5.4 ± 2.5*	4.0 ± 2.0
T_c_ (ms)	34.4 ± 16.5	34.7 ± 17.4	41.0 ± 21.7	40.2 ± 22.7
T_d_ (ms)	23.2 ± 3.2*	21.2 ± 2.7	24.0 ± 4.2*	21.8 ± 2.7
T_r_ (ms)	75.9 ± 44.3	108.2 ± 120.7	70 ± 50.8	83.4 ± 80.6
T_s_ (ms)	202.7 ± 70.6	264.4 ± 177.2	192.7 ± 87.8	219.8 ± 98.3
V_c_ (mm/s)	76.2 ± 25.1*	57.2 ± 24.3	74.8 ± 34.9	60.3 ± 26.8
Amplitude (mA)	85.6 ± 18.5	87.9 ± 16.2	89.4 ± 13.6	91.2 ± 14.7

Data are presented as means ± standard deviations

(*) = significant difference between BF-mid and BF-distal (measurement sites).

### Measurement conditions

No significant differences in the TMG-derived parameters were revealed for the tested conditions (fixed vs. non-fixed) at either measurement site (BF-mid: T_d_, *p* = 0.5; T_c_, *p* = 0.3, D_m_, *p* = 1.0, V_c_, *p* = 0.2; BF-distal: T_d_, *p* = 0.2, T_c_, *p* = 0.3, D_m_, *p* = 0.3, V_c_, *p* = 0.5; Table 2).

**Fig 2:**
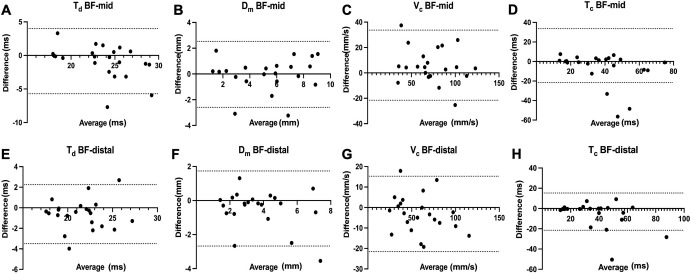
Limits of agreement analysis between the fixed and non-fixed conditions across the TMG-derived parameters. A = T_d_ at BF-mid, B = D_m_ at BF-mid, C = V_c_ at BF-mid, D = T_c_ at BF-mid, E = T_d_ at BF-distal, F = D_m_ at BF-distal, G = V_c_ at BF-distal, H = T_c_ at BF-distal.

Pearson’s correlation revealed a significant positive relationship between T_d_ in the fixed and non-fixed conditions at both measurement sites (BF-mid: *r* = 0.8, *p* < 0.001; BF-distal: *r* = 0.9, *p* < 0.001). Bland-Altman analysis for T_d_ showed a mean bias of -0.8 ± 2.5 ms (LOA: -5.7 to 4.0 ms; slope coefficient = -0.3, *p* = 0.04) at BF-mid and -0.6 ± 1.5 ms (LOA: -3.5 to 2.3 ms) at BF-distal, respectively (Fig 2). A significant positive correlation was also found between D_m_ in the fixed and non-fixed condition (BF-mid: *r* = 0.9, *p* < 0.001; BF-distal: *r* = 0.8, *p* < 0.001). Furthermore, Bland-Altman analysis showed a mean bias of -0.04 ± 1.3 mm (LOA: -2.6 to 2.5 mm) and -0.5 ± 1.1 mm (LOA: -2.7 to 1.7 mm) between BF-mid and BF-distal, respectively. Significant positive correlations were also revealed for T_c_ between both conditions (BF-mid: *r* = 0.6, *p* = 0.002; BF-distal: *r* = 0.8, *p* < 0.001). Bland-Altman analysis for T_c_ showed a mean bias of -6.6 ± 17.2 ms (LOA: -40.2 to 27.0 ms) at BF-mid and -5.5 ± 13.4 ms (LOA: -31.8 to 20.8 ms) at BF-distal. Finally, V_c_ correlated significantly between conditions at BF-mid (*r* = 0.9, *p* < 0.001) and BF-distal (*r* = 0.9, *p* < 0.001). Bland-Altman analysis showed a mean bias of 6.0 ± 14.1 mm/s (LOA: -21.6 to 33.7 mm/s) and -3.1 ± 9.4 mm/s (LOA: -21.5 to 15.3 mm/s) between BF-mid and BF-distal, respectively.

### Measurement sites

**Fig 3:**
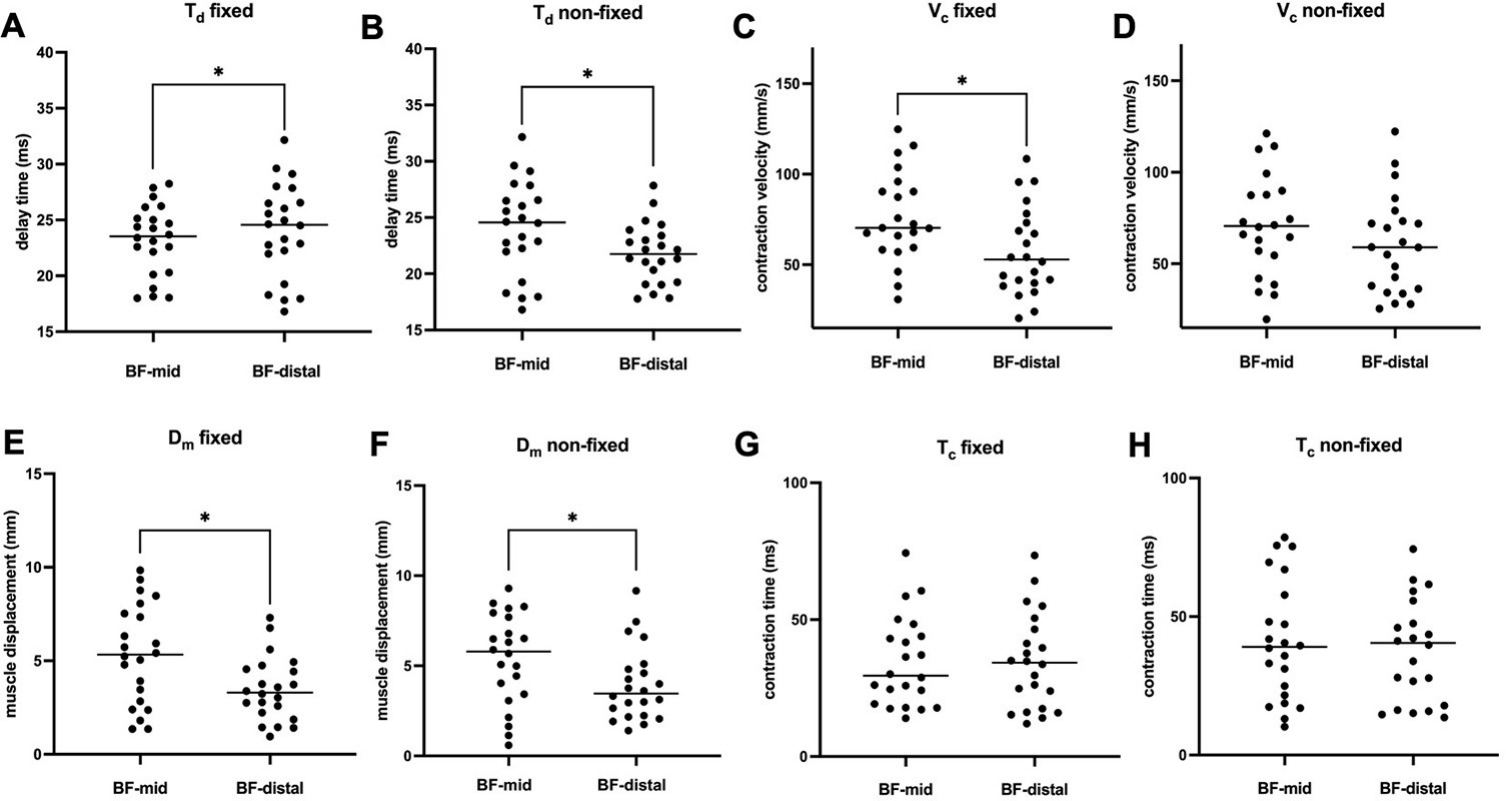
Differences between the TMG-derived parameters for measurement sites on the m. biceps femoris. A = differences in T_d_ for the fixed condition; B = differences for T_d_ in the non-fixed condition; C = differences in V_c_ in the fixed condition; D = differences in D_m_ in the fixed condition; E = differences in D_m_ in the non-fixed condition. [*] = significant difference between the measurement sites.

Sensor location (BF-mid vs. BF-distal) affected the contractile properties significantly across the conditions (fixed vs. non-fixed) (Fig 3). For the fixed condition, T_d_ differed significantly between the measurement locations by -2.0 ms (95% CI: -3.3 and -0.7 ms), *p* = 0.002 (*d*_*z*_ = 0.9), which is in line with the non-fixed condition (mean difference of -2.2 ms, 95% CI: -4.0 and -0.5 ms; *p* = 0.008, *d*_*z*_ = 0.7). In both conditions, T_d_ was greater at BF-mid compared to BF-distal (fixed: 23.2 ± 3.2 ms vs. 21.2 ± 2.7 ms; non-fixed: 24.0 ± 4.2 ms vs. 21.8 ± 2.7 ms). D_m_ in the fixed condition differed significantly by -1.9 mm (95% CI: -3.2 and -0.5 mm), *p* = 0.005, *d*_*z*_ = 0.8), which is in line with the non-fixed condition (mean change of -1.4 mm, 95% CI: -2.7 and -0.1 mm; *p* = 0.03, *d*_*z*_ = 0.6). D_m_ was larger at BF-mid compared to BF-distal (fixed: 5.3 ± 2.7 mm vs. 3.5 ± 1.7 mm; non-fixed: 5.4 ± 2.5 mm vs. 4.0 ± 2.0 mm). For T_c_, no significant differences between the measurement sites were found (fixed, *p* = 1.0; non-fixed, *p* = 1.0). V_c_ only revealed significant differences between the measurement sites in the fixed condition with a mean difference of -19.5 mm/s (95% CI: -44.8 and -2.5 mm/s, *p* = 0.02, *d*_*z*_ = 0.7). Likewise, V_c_ revealed larger values at BF-mid (V_c_: 76.7 ± 25.1 mm/s vs. 57.2 ± 24.3 mm/s).

## Discussion

The primary goals of the present study were to investigate whether (1) sensor location and (2) lower leg fixation affect the muscle contractility of the BF. The findings of our study imply that sensor location highly affected the TMG-derived muscle contractility. Accordingly, our first hypothesis can be confirmed. Our results indicate faster muscle responsiveness at BF-distal (smaller T_d_) and a faster and larger muscle displacement at BF-mid (larger D_m_ and V_c_). Contrarily, our second hypothesis must be rejected as the lower leg fixation did not alter muscle contractility of the BF.

TMG has been described as a method to measure the radial muscle belly displacement during an isometric twitch contraction [3]. As previous research has reported changes in the joint angle [18, 19], it seems reasonable to fixate the subject’s extremity during the measurement to prevent unwanted limb movement accompanied by changes in the muscle length. In line with this reasoning, several previous studies focusing on the reliability [27] and validity of the TMG method [28–30] also used straps to fixate the subjects’ limbs to ensure isometric conditions during the measurement. Our findings indicate that fixation of the lower leg slightly above the ankle did not affect the TMG-derived parameters when carried out on various sites on the BF. Bland-Altman analysis revealed nearly ideal agreement in T_d_, D_m_, and V_c_, suggesting null effects between the conditions at the BF. These findings are beneficial for researchers and practitioners who use TMG on the BFs regularly and rely on standardized results, as they can now exclude lower leg movement as a possible interference. For the first time, our results show that the lower leg movement does not affect the validity of TMG measurements on the BF. This can be important for practitioners, who conduct measurements in the field and do not have the possibility to fixate the respective extremity. It is important to note that these findings cannot be extrapolated to other muscles due to differences in muscle characteristics such as the pennation angle or fiber distribution.

Collectively, beyond D_m_, T_c_ and V_c_, which are frequently used in TMG research, this paper has underlined the potential role of T_d_ in assessing skeletal muscle contractility. The results of our study revealed that T_d_, e.g., muscle responsiveness, was significantly faster at BF-distal than at BF-mid in both measurement conditions. Two possible reasons emerge as explanations for this. First, T_d_ is directly affected by the extent of D_m_, which was also smaller at BF-distal than at BF-mid. However, the faster muscle responsiveness could also be explained by the BF-distal being closer to the muscle tendon junction. One of the most important functions of the musculotendinous unit in sports is its ability to produce explosive concentric contractions [31]. In high performance sports, this ability is usually preceded by an eccentric contraction, resulting in the established strength-shortening cycle, which is indispensable to rapid movements such as sprinting and jumps [31]. The ability of an athlete to utilize the strength-shortening cycle efficiently is expressed as the reactive-strength index and is usually calculated by dividing the jump height by the ground contact time during a jumping exercise [32]. While further research is required, TMG could supplement measurements that investigate an athlete’s reactive strength and provide information about the muscular conditions of the hamstrings. Confirmatory, Gil et al. [33] reported a moderate association between D_m_ of the BF and the reactive strength index. Based on our results, the association between TMG parameters and reactive strength could change when performing the TMG measurements more distal to the knee joint. Accordingly, our findings can help researchers and practitioners to gain a holistic understanding of an athlete’s muscular characteristics and could result in an advanced optimization of physical performance. Further, a recent publication by Dordevic et al. [34] has shown that TMG is sensitive to discriminate between injured and non-injured BF muscles via analysis of the contraction time. The results of our study might help gaining further information about the exact injury location within the muscle. TMG has also been used to assess inter-limb differences in female soccer players [35], with the authors concluding that more in-depth information about TMG measurements are necessary to correctly interpret the results. Lastly, TMG-derived contraction velocity appears to be a sensible marker to detect and interpret acute changes on performance of elite team sports athletes [36].

The present data demonstrated higher values of D_m_ at BF-mid compared to BF-distal across the conditions, as well as a faster muscle displacement at BF-mid (V_c_). Our results are consistent with the findings of a previous study which found reductions in the D_m_ of the m. rectus femoris and m. gastrocnemius medialis, ranging from 3% to 4%, when the sensor was moved 1 cm distally from the reference measuring site [37]. Interestingly, this phenomenon could be muscle-specific since, in the same study, D_m_ of the m. gastrocnemius lateralis did not increase significantly, i.e., by 1%, when measured distally from the reference site [37]. In contrast to these results, we found statistically significant reductions in D_m_ of 21% in both conditions, when measured 10 cm distal to the reference measuring site. However, these results should be interpreted with a certain amount of caution as our data exposed a large standard deviation (± 54% for fixed; ± 35% for non-fixed) in both conditions. A possible reason for this could be inter-individual differences in subcutaneous fat tissue, which has been reported to possibly affect mechanomyography signals [38]. Even though there were no significant differences in baseline body fat, the body fat percentage varied between 7.8% and 21.8%. It should be mentioned though, that there are also studies, who found no effect of subcutaneous fat on TMG measurements [39]. Another reason could be the differences in the training regimen of our subjects, as Loturco et al. [40] reported different outcomes in D_m_ and T_c_ for power and endurance athletes.

The difference in the magnitude of the relative reduction in D_m_ between our results and those of John et al. [37] is consistent with the findings by Simunic et al. [30]. In this study, three displacement sensors were used in parallel to determine the spatial error distribution of D_m_ and T_c_ in five different muscles [30]; their results showed an increase in the relative difference of D_m_ and T_c_ with increasing distance from a predetermined reference measuring site in the distal, proximal, medial and lateral directions [30].

In summary, while TMG is regularly used for load monitoring [41] and injury prevention in elite sports [37, 38], there is still a lack of the standardization and systematic use of TMG in several sports and research areas. Previous studies have shown that sensor placement on the muscle belly affects the D_m_ and T_c_ of different muscles of the trunk and the upper and lower extremities [17, 30, 31]. Furthermore, research indicates that muscle physiology, e.g., muscle damage, differs within a muscle head following load exposure [42], indicating that the sensor location could help researchers and practitioners with the evaluation of muscle damage variations within a single muscle. The fact that our study showed a significant influence of the sensor position on the TMG-derived parameters indicates that an adequate interpretation of data from different muscle regions requires knowledge of the intra-individual baseline values. Our findings are important for researchers and practitioners in different fields to optimize their measurement routines and to tailor the measurement conduction regarding the individual assessment goals. For example, while using the same measurement protocol, the usage of TMG for a rehab trainer in high performance sports might be different compared to a sports scientist aiming to investigate changes in muscle contractility during a prolonged caloric deficit in participants. However, this study is not free of limitations. As we only recruited male participants, the external validity of our results is hampered. Further, our results revealed a relatively large standard deviation of certain TMG parameters. This shows how variable TMG measurements can be, which emphasizes the need to detect and appropriately interpret possible confounders even more.

## Conclusion

The current study aimed to investigate whether sensor location and lower leg fixation affected TMG measurements performed on the BF. While no influence of lower leg fixation was observed, sensor location affected the muscle contractility parameters significantly. D_m_ and V_c_ exposed higher values at 50% length of the muscle, while T_d_ was shorter at the measurement site 10 cm distal of the 50% muscle length. These findings indicate that muscle contractility, as expected, is more pronounced at the muscle belly and that muscle responsiveness is faster towards the musculotendinous junction. Collectively, beyond D_m_, T_c_ and V_c_, which are frequently used in TMG research, this paper has underlined the potential role of T_d_ in assessing skeletal muscle contractility. Our findings may help researchers and practitioners to create tailored study protocols and measurement procedures in compliance with the individual goals of the TMG measurements and to allow the adequate interpretation of the TMG-derived parameters.
